# Персонифицированный подход к терапии ожирения на основе доказательных данных и клинических алгоритмов: семаглутид или тирзепатид

**DOI:** 10.14341/probl13677

**Published:** 2025-12-02

**Authors:** Е. А. Трошина, М. Б. Анциферов, А. С. Аметов, Г. Р. Галстян, Т. Н. Маркова, Т. И. Романцова, Н. В. Мазурина, О. М. Котешкова

**Affiliations:** Национальный медицинский исследовательский центр эндокринологии им. акад. И.И. ДедоваРоссия; Endocrinology Research CentreRussian Federation; Эндокринологический диспансер г. МосквыРоссия; Endocrinological DispensaryRussian Federation; Российская медицинская академия непрерывного профессионального образованияРоссия; Russian Medical Academy of Continuing Professional EducationRussian Federation; Российский университет медициныРоссия; Russian University of MedicineRussian Federation

**Keywords:** ожирение, семаглутид, тирзепатид, obesity, semaglutide, tirzepatide

## Abstract

26 июня 2025 года в Москве прошло заседание экспертной рабочей группы. В центре обсуждения были вопросы персонализированного применения тирзепатида («Тирзетта®») и семаглутида («Велгия ЭКО®») у пациентов с избыточной массой тела, ожирением и сахарным диабетом 2 типа. По итогам встречи была поставлена задача разработать консенсусный алгоритм назначения этих препаратов для их эффективного использования в российской клинической практике.

## Abstract (en)

On June 26, 2025, a meeting of the expert working group was held in Moscow. The discussion focused on the personalized approach of tirzepatide («Tirzetta®») and semaglutide («Velgia Eco®») in patients with excess body weight, obesity, and type 2 diabetes. Following the meeting, the objective was set to develop a consensus-based algorithm for prescribing these drugs to ensure their effective use in Russian clinical practice.

Эпидемическое распространение ожирения и сахарного диабета 2 типа (СД2), ассоциированных с высоким кардиометаболическим риском, представляет собой одну из наиболее серьезных угроз общественному здоровью в XXI веке. Традиционные терапевтические подходы демонстрируют ограниченную эффективность в отношении снижения массы тела и сердечно-сосудистых исходов в долгосрочной перспективе. Появление и внедрение в клиническую практику препаратов на основе механизма действия инкретинов ознаменовало новую эру в лечении метаболических заболеваний. Агонисты рецепторов глюкагоноподобного пептида-1 (арГПП-1) и, впоследствии двойные и мультиагонисты, оказывают комплексное воздействие на ключевые патофизиологические звенья СД2 и ожирения. Эти препараты не только эффективно контролируют уровень глюкозы и массу тела, но и демонстрируют плейотропные эффекты в отношении сердечно-сосудистой системы, неалкогольной жировой болезни печени (НАЖБП), хронической болезни почек (ХБП), синдрома обструктивного апноэ сна (СОАС) и остеоартрита (ОСА), что делает их важнейшей составляющей современной персонализированной терапии [1][2].

26 июня 2025 г. в Москве состоялось совещание рабочей группы экспертов на тему «Обсуждение персонификации терапии тирзепатидом (Тирзетта) и семаглутидом (Велгия ЭКО) и подготовка алгоритма их назначения пациентам с избыточным весом или ожирением, в том числе с СД 2 типа». Целью совещания была выработка консенсусной позиции по оптимальному применению агонистов инкретиновых рецепторов в реальной клинической практике с учетом актуальных международных и отечественных данных. История создания этих препаратов началась с изучения редкой ядовитой ящерицы Gila Monster (Heloderma suspectum). Это животное, обитающее в пустынях Аризоны вблизи реки Gila, проводит до 90% времени под землей и питается очень редко, что натолкнуло ученых на мысль об уникальных механизмах регуляции метаболизма [3][4]. В слюне этой ящерицы был обнаружен пептид эксендин-4, ставший прототипом для разработки первого агониста рецепторов ГПП-1 [5]. Как было отмечено в одном из выпусков журнала “Diabetologia”, препараты на основе эффектов инкретинов стали своего рода антидотом для множества заболеваний, связанных с диабетом и ожирением [6].

Несмотря на то, что оба препарата относятся к одному классу, их молекулярные механизмы и, как следствие клинические эффекты, имеют существенные различия. Семаглутид — агонист единственного рецептора, глюкагоноподобного пептида-1 (ГПП-1), с периодом полувыведения около недели (160 часов). Тирзепатид — это двойной агонист, воздействующий как на рецепторы ГПП-1, так и на рецепторы глюкозозависимого инсулинотропного полипептида (ГИП). Период полувыведения тирзепатида несколько короче — порядка 5 дней (116,7 часа). Аффинность тирзепатида к рецептору ГИП сопоставима с аффинностью нативного гормона, аффинность к рецептору ГПП-1 в 5 раз слабее. Рецепторы инкретинов относятся к классическим рецепторам, сопряженным с G-белками [7][8]. Рецепторы содержат большой внеклеточный N-концевой домен, отвечающий за связывание лиганда, 7 трансмембранных α-спиралей и внутриклеточный С-концевой домен, который взаимодействует с G-белками и другими сигнальными молекулами. При связывании с лигандом рецептор претерпевает конформационные изменения, которые активируют внутриклеточные сигнальные каскады за счет сопряжения со стимулирующим G-белком, что приводит к активации аденилатциклазы и последующему повышению уровня циклического аденозинмонофосфата (цАМФ), что в итоге приводит к соответствующему клеточному ответу (повышению секреции инсулина в β-клетках, высвобождению нейротрансмиттеров в нейронах и т.д.). Помимо повышения ПКА, взаимодействие рецепторов с лигандом сопровождается рекрутированием β-аррестина, который способствует интернализации рецепторов, уменьшению их количества на клеточной мембране. Вместе с фрагментом мембраны рецептор погружается в цитоплазму клетки, формируя эндосомальный компартмент. Некоторые эндосомальные компартменты вновь возвращаются к мембране, в то время как другие сливаются с лизосомами. Семаглутид является сбалансированным лигандом по отношению к рецепторам ГПП-1. Его взаимодействие с рецепторами сопровождается как повышением ПКА, так и рекрутированием β-аррестина. Тирзепатид — сбалансированный лиганд по отношению к рецепторам ГИП, но смещенный лиганд по отношению к рецепторам ГПП-1: активация внутриклеточных сигнальных путей сопровождается минимальным рекрутированием β-аррестина и, соответственно, снижением десенситизации рецепторов ГПП-1. Эти особенности фармакодинамики во многом объясняют более выраженное влияние тирзепатида на массу тела и углеводный обмен по сравнению с арГПП-1 [9][10].

Рецепторы инкретинов расположены на всех трех основных типах островковых типах клеток: альфа, бета, дельта. Оба препарата усиливают секрецию инсулина и соматостатина, подавляют апоптоз бета-клеток поджелудочной железы. Семаглутид подавляет секрецию глюкагона. Тирзепатид в изолированных островковых клетках увеличивает секрецию глюкагона. У больных СД2 на фоне лечения препаратом происходит постепенное снижение содержания гормона [11][12][13].

## ВЛИЯНИЕ НА ЦЕНТРАЛЬНУЮ НЕРВНУЮ СИСТЕМУ И КОНТРОЛЬ АППЕТИТА

Рецепторы инкретинов расположены в ключевых зонах мозга, отвечающих за контроль аппетита и пищевого поведения, включая гипоталамус, ствол мозга и систему вознаграждения, что объясняет их комплексное анорексигенное действие. Семаглутид воздействует на более обширные зоны мозга, чем его предшественники. Кроме того, препарат повышает продукцию пролактин- релизинг-гормона (мощного анорексигенного нейромедиатора) и тирозингидроксилазы, участвующей в синтезе дофамина [14]. Тирзепатид реализует более выраженный эффект подавления аппетита путем активации обоих видов инкретиновых рецепторов на уровне гипоталамуса и ствола мозга, а также способствуя повышению проницаемости гематоэнцефалического барьера для периферических анорексигенных гормонов [15][16]. Оба препарата снижают тягу к сладкой, жирной и соленой пище и показывают положительные результаты в лечении различных форм зависимостей, в том числе алкогольной [17].

## ПЕРЕНОСИМОСТЬ: ПОЧЕМУ ТИРЗЕПАТИД В МЕНЬШЕЙ СТЕПЕНИ ВЫЗЫВАЕТ ТОШНОТУ?

Одним из главных преимуществ тирзепатида является его лучшая переносимость в отношении возникновения тошноты и рвоты. Механизм этого обусловлен двойном действием на «рвотный центр» в стволе мозга (area postrema).

Нейроны, экспрессирующие рецепторы обоих инкретинов, относительно малочисленны. В большинстве случаев рецепторы ГИП и ГПП-1 локализованы в различных популяциях нейронов. Эти анатомические особенности свидетельствуют о том, что агонисты ГПП-1р и ГИПр влияют на разные нейронные цепи [18]. В области area postrema подавляющее большинство нейронов, экспрессирующих рецепторы ГИП — ГАМКергические (ингибирующие), а большинство ГПП-1р-содержащих нейронов — глутаматергические (возбуждающие). Активация рецепторов ГПП-1 (как в случае семаглутида) возбуждает эти нейроны, вызывая не только снижение аппетита, но и тошноту. Под действием тирзепатида активация нейронов, экспрессирующих рецепторы ГИП на уровне area postrema, сопровождается выработкой гамма-амино-масляной кислоты (ГАМК), что подавляет активность нейронов, содержащих рецепторы ГПП-1, тем самым значительно снижая риск развития тошноты и рвоты [19]. Кроме того, тирзепатид подавляет эффекты пептида YY на уровне area postrema (попытки разработать моноагонисты рецепторов пептида YY для фармакотерапии ожирения не увенчались успехом вследствие выраженной тошноты и рвоты на фоне их применения) [20].

## ЖЕЛУДОЧНО-КИШЕЧНЫЙ ТРАКТ И ЛЕКАРСТВЕННЫЕ ВЗАИМОДЕЙСТВИЯ

Степень замедления моторики желудка у тирзепатида и семаглутида сопоставимы (исследования проведены у животных, у пациентов с СД2, с ожирением) [21]. Замедление опорожнения желудка на фоне обоих препаратов относится к числу их периферических механизмов снижения массы тела. Однако этот эффект требует внимания при выполнении эндоскопии либо оперативных вмешательств вследствие повышения риска регургитации и легочной аспирации. Анестезиологи рекомендуют отменять препараты минимум за 1 неделю до планируемых вмешательств, однако этот период может оказаться недостаточным для полного восстановления функции желудка. Оптимальная продолжительность прекращения приема GLP-1RA для снижения риска аспирации в настоящее время неизвестна, тщательные исследования в этой области продолжаются [22]. Изменение моторной функции желудочно-кишечного тракта может влиять на фармакокинетику других применяемых пациентом препаратов. Клинически значимое снижение абсорбции пероральных контрацептивов подтверждено на фоне применения тирзепатида, L-тироксина — на фоне терапии семаглутидом [23].

## ВОСПАЛЕНИЕ И ЖИРОВАЯ ТКАНЬ

Хроническое воспаление — ключевой фактор развития осложнений ожирения. Как семаглутид, так и тирзепатид, эффективно подавляют воспаление. Механизмы, посредством которых препараты влияют на воспалительные процессы, имеют существенные отличия. Рецепторы ГПП-1 на клетках иммунной системы экспрессированы достаточно скудно. Семаглутид непосредственно снижает локальное воспаление лишь в ЖКТ путем активации Т-лимфоцитов. Системное воспаление на фоне семаглутида снижается путем активации центральных рецепторов ГПП-1 с участием адренергических и опиоидных рецепторов. Тирзепатид действует напрямую на иммунные клетки, которые несут на своей поверхности большое количество рецепторов ГИП [24].

В жировой ткани рецепторы ГПП-1 до настоящего времени не идентифицированы. Преимущественно посредством вовлечения симпатической нервной системы в белой жировой ткани семаглутид снижает воспаление, активирует липолиз, захват липидов, снижает содержание висцерального эктопического жира, повышает конверсию белых адипоцитов в бежевые и термогенез [25]. Рецепторы ГИП экспрессируются на многих клетках жировой ткани, включая бурые и бежевые адипоциты, макрофаги, перициты, мезотелий [12][26]. Тирзепатид оказывает прямое влияние на жировую ткань. Активация рецепторов ГИП под действием тирзепатида в белой жировой ткани способствует повышению кровотока (транспорт нутриентов и кислорода), чувствительности адипоцитов к инсулину, захвата глюкозы и СЖК, липогенезу de novo. Повышение термогенеза под действием тирзепатида реализуется на основе не только классических механизмов (повышение экспрессии белка UCP-1, разобщающего окислительное фосфорилирование), но и путем UCP-1-независимого футильного кальциевого цикла [27].

## ЗАЩИТА ОРГАНОВ-МИШЕНЕЙ

Сердце и сосуды. Препараты обладают мощным кардиопротективным действием, снижая воспаление, улучшая сократимость миокарда и стабилизируя атеросклеротические бляшки. Семаглутид одобрен для снижения сердечно-сосудистых рисков [28].

Почки. Семаглутид снижает риск прогрессирования хронической болезни почек у пациентов с СД и одобрен для применения в этой группе пациентов. Тирзепатид также демонстрирует впечатляющие результаты в этой области [29].

Мозг. Оба препарата оказывают нейропротективное действие, снижая нейровоспаление и накопление амилоида, что открывает перспективы для лечения нейродегенеративных заболеваний [30].

Легкие. Тирзепатид рекомендован для лечения синдрома обструктивного апноэ сна, что является большим преимуществом для пациентов с ожирением [31].

Глубокое понимание уникальных механизмов действия семаглутида и тирзепатида является ключом к максимально эффективному и безопасному применению этих инновационных препаратов в клинической практике. В свою очередь, анализ данных имеющихся рандомизированных клинических исследований (РКИ) позволяет сформировать практические подходы к выбору терапии: от общих установок к персонализированной, доказательной медицине.

## ОТ КИЛОГРАММОВ К КЛИНИЧЕСКИМ ИСХОДАМ: ПОСТАНОВКА ЦЕЛЕЙ ТЕРАПИИ

В лечении ожирения крайне важно определять индивидуальные цели для каждого пациента, выходя за рамки простого снижения веса. Была предложена четкая градация терапевтических целей (табл. 1):

- снижение веса на 5–10%: уменьшение симптоматики и улучшение качества жизни;

- снижение веса более чем на 15%: такой результат необходим для достижения более амбициозных целей, таких как ремиссия сопутствующих заболеваний, снижение смертности и предотвращение тяжелых осложнений, включая сердечную недостаточность и другие сердечно-сосудистые события [32].

**Figure:**
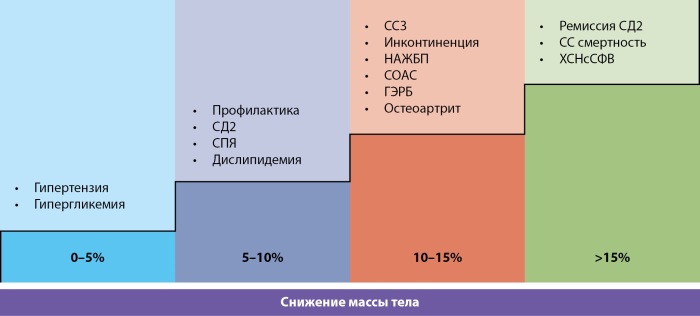
Таблица 1. Улучшение течения сопутствующих состояний зависит от процента снижения массы тела

## ПРЯМОЕ СРАВНЕНИЕ ТИРЗЕПАТИДА И СЕМАГЛУТИДА В ИССЛЕДОВАНИИ SURMOUNT-5

Ключевым для понимания эффективности стало исследование SURMOUNT-5, в котором напрямую сравнивались максимальные дозы тирзепатида (15 мг) и семаглутида (2,4 мг) у пациентов с ожирением, но без СД. Продолжительность исследования составила 72 недели (табл. 2).

**Table:** Таблица 2. Surmount-5: ожирение и избыточная масса тела Цель терапии: профилактика развития осложнений Показатель эффективности: % снижения массы тела, ↓окружности талии Значения представлены в виде расчетной разницы в процентных пунктах между группами, за исключением категорий снижения веса, которые составляют не менее 10%, 15%, 20%, 25%, и 30%, которые указаны как относительный риск. Относительный риск был рассчитан с использованием G-методы расчета на основе логистической регрессии. P<0,001 для всех первичных и ключевых вторичных конечных точек.

Параметр	Семаглутид 2,4 мг	Тирзепатид 15 мг
Средняя потеря веса	-13,7% (95% ДИ: -14,9 до -12,6)	-20,2% (95% ДИ: -21,4 до -19,1)
Абсолютная потеря веса	-15,0 кг (95% ДИ: -16,3 до -13,7)	-22,8 кг (95% ДИ: -24,1 до -21,5)
Уменьшение окружности талии	-13,0 см (95% ДИ: -14,3 до -11,7)	-18,4 см (95% ДИ: -19,6 до -17,2)
Снижение ИМТ	-6,0 кг/м²	-8,5 кг/м²
Достижение целевых показателей потери веса
≥10%	60,5%	81,6%
≥15%	40,1%	64,6%
≥20%	27,3%	48,4%
≥25%	16,1%	31,6%
≥30%	6,9%	19,7%

Эффективность: тирзепатид продемонстрировал статистически значимое преимущество по всем ключевым показателям снижения веса, включая среднюю и абсолютную потерю массы тела, а также уменьшение окружности талии. Так, 81% пациентов на тирзепатиде и 60% на семаглутиде достигли снижения веса на 10% и более, что является клинически значимым результатом для прогноза.

Безопасность и переносимость: общая частота нежелательных явлений была сопоставимой между двумя препаратами. Наиболее частыми побочными эффектами со стороны ЖКТ были тошнота (около 44% в обеих группах), запор (28%) и диарея (23%). Важно отметить, что частота серьезных нежелательных явлений, приведших к прекращению терапии (включая панкреатит и сердечно-сосудистые события), была очень низкой и не различалась между группами [33].

## ВЛИЯНИЕ НА СОПУТСТВУЮЩИЕ ЗАБОЛЕВАНИЯ: ДАННЫЕ КЛИНИЧЕСКИХ ИССЛЕДОВАНИЙ

## Сердечно-сосудистые риски

Семаглутид. Исследование SELECT стало прорывным, доказав, что препарат в дозе 2,4 мг снижает риск серьезных сердечно-сосудистых событий (MACE) на 20% у пациентов с ожирением и установленными ССЗ, но без СД (табл. 3). Эффект наблюдался практически с самого начала терапии, что указывает на прямые кардиопротективные механизмы, а не только как на результат снижения веса [34]. Данные реальной практики, полученные в исследования SCORE, оказались еще более впечатляющими, показав снижение общей смертности на 86% [35]. Эти результаты позволили зарегистрировать новое показание для семаглутида — снижение риска сердечно-сосудистых событий у пациентов с ожирением.

**Table:** Таблица 3. Семаглутид/Тирзепатид: влияние на течение АССЗ Цель терапии: снижение рисков сердечно-сосудистых катастроф

Параметр	Семаглутид 2,4 мг (SELECT)	Тирзепатид 15 мг (SURMOUNT-MMO)
MACE	-20%HR, 0,80 (95% ДИ, 0,72–0,90) P<0,001 for superiority	В процессе изучения
Смерть от всех причин	-19%HR, 0,81 (95% ДИ, 0,71–0,93)
Нефатальный инфаркт миокарда	-28%HR, 0,72 (95% ДИ, 0,61 to 0,85)
СС смертность	-15%HR, 0,85 (95% ДИ, 0,71–1,01) P=0,07
Развитие и усугубление ХБП	-22%HR, 0,78 (95% ДИ, 0,63 to 0,96)
Развитие и усугубление ХСН	-18%HR, 0,82 (95% ДИ, 0,71–0,96)

Тирзепатид. Аналогичное исследование SURMOUNT-MMO для препарата еще продолжается. Ожидается, что его результаты также будут положительными.

## ВЛИЯНИЕ НА ХРОНИЧЕСКУЮ СЕРДЕЧНУЮ НЕДОСТАТОЧНОСТЬ С СОХРАННОЙ ФРАКЦИЕЙ ВЫБРОСА (ХСННФВ)

Тирзепатид 15 мг. В исследовании SUMMIT препарат продемонстрировал выраженный эффект, снизив риск госпитализаций по поводу ХСН на 38% [36].

Семаглутид 2,4 мг. Показано значительное улучшение показателей по шкалам оценки качества жизни и тестам с шестиминутной ходьбой, а также снижение уровня С-реактивного белка [37]. В исследовании реальной практики SCORE снижение риска СН достигло 54% (табл. 4) [35].

**Table:** Таблица 4. Семаглутид/Тирзепатид: влияние лечения на ХСНСФВ у пациентов с ожирением Цель терапии: ↓числа госпитализаций, улучшение функциональной активности больных

Параметр	Семаглутид 2,4 мг (STEP-HFpEF)	Тирзепатид 15 мг (SUMMIT)
Продолжительность исследования	52 недели	104 недели
Снижение риска госпитализаций и ухудшения ХСН	STEP-HFpEF не оценивали в рамках КИ; SCORE (120 недель): -54% HR 0,46 (95% ДИ: 0,29–0,73, p<0,05)	-38% HR 0,62 (95% ДИ: 0,41–0,95; p=0,026)
Улучшение KCCQ-CSS	+7,8 баллов (p<0,001)	+6,9 баллов (p<0,001)
Улучшение 6-мин теста	+21,5 м (разница: +20,3 м; p<0,001)	+26,0 м (разница: +18,3 м; p<0,001)
Снижение вчСрб	-43,5% (разница: -36,2%, p<0,001)	-38,8% (разница: -34,9%)

## СИНДРОМ ОБСТРУКТИВНОГО АПНОЭ СНА (СОАС)

Тирзепатид является единственным препаратом, у которого есть завершенное РКИ и официальное одобрение для лечения СОАС средней и тяжелой степени тяжести у взрослых с ожирением в сочетании с низкокалорийной диетой и повышенной физической активностью. Исследование показало значительное снижение количества приступов апноэ/гипопноэ в час (табл. 5) [38].

**Table:** Таблица 5. Семаглутид/Тирзепатид: влияние лечения на течение синдрома обструктивного апноэ сна у пациентов с ожирением Цель терапии: изменение индекса апноэ–гипопноэ (AHI, количество приступов апноэ и гипопноэ в течение часа сна) по сравнению с исходным уровнем

Параметр	Семаглутид 2,4 мг	Тирзепатид 15 мг (SURMOUNT-OSA)
Характеристика пациентов	Не изучался	ИМТ: не менее 30 кг/м² (в среднем 39 кг/м²) Индекс апноэ-гипопноэ: не менее 15 событий за час (в среднем 50) Пациенты без СД1 и СД2
Изменение индекса апноэ–гипопноэ	-29 эпизодов 95% ДИ, −33,2 to −25,4, P<0,001

## ОСТЕОАРТРИТ КОЛЕННОГО СУСТАВА

Семаглутид имеет доказательную базу по положительному влиянию на остеоартрит, что связано не только со снижением нагрузки на суставы, но и с прямым противовоспалительным действием. В ходе клинического исследования STEP-9 продемонстрировано значимое снижение болевого синдрома и улучшение функциональной активности по шкале WOMAC у пациентов с ожирением и остеоартритом коленного сустава (табл. 6) [39].

**Table:** Таблица 6. Семаглутид/Тирзепатид: влияние лечения на течение остеоартрита коленного сустава Цель терапии: снижение болевого синдрома, улучшение функциональной подвижности

Параметр	Семаглутид 2,4 мг (STEP-9)	Тирзепатид 15 мг
Характеристика пациентов	407 пациентов, возраст 56 лет, ИМТ 40,3, ср. значение по шкале боли WOMAC 70,9	Не изучался
Снижение болевого синдрома по шкале WOMAC	-41,7
Улучшение функциональной подвижности за счет снижения скованности по шкале WOMAC	-41,5

## НЕАЛКОГОЛЬНАЯ/МЕТАБОЛИЧЕСКИ АССОЦИИРОВАННАЯ ЖИРОВАЯ БОЛЕЗНЬ ПЕЧЕНИ (НАЖБП/МАЖБП)

Исследование 2 фазы SYNERGY-NASH продемонстрировало потенциальный положительный эффект при применении тирзепатида 15 мг и способствовало разрешению стеатогепатита у 62% пациентов и уменьшению стадии фиброза у 51% из них. На сегодняшний день данный препарат изучается в клинических испытаниях 3-й фазы [40] (табл. 7).

**Table:** Таблица 7. Семаглутид/Тирзепатид: влияние на развитие и течение НАЖБП Цель терапии: разрешение стеатогепатоза и уменьшение стадии фиброза

Параметр	Семаглутид 2,4 мг (ESSENCE 3 фаза, промежуточные результаты)	Тирзепатид (SYNERGY-NASH 2 фаза)
Разрешение стеатогепатоза без ухудшения степени фиброза печени	62,9%95% ДИ: 21,3–36,5, p<0,0001	62%95% ДИ: 37 to 69, p<0,001
Уменьшение стадии фиброза, без прогрессирования стеатогепатоза	37%95% ДИ: 7,5–21,4, p<0,0001	51%95% ДИ: 1 to 42, p<0,001

Применение Семаглутида 2,4 мг в исследовании ESSENCE способствовало разрешению стеатогепатита у 62,9% пациентов и уменьшению стадии фиброза у 37% [41]. В 2025 г. FDA одобрило применение семаглутида для лечения метаболически ассоциированного стеатогепатита (MASH), также известного как неалкогольная жировая болезнь печени, у взрослых с фиброзом средней и высокой степени выраженности.

## ПРЕДИАБЕТ / ПРОФИЛАКТИКА СД2

Семаглутид 2,4 мг и тирзепатид 15 мг продемонстрировали нормализацию гликемии у 81% и 95% пациентов соответственно, что свидетельствует об эффективной профилактике развития СД2 у пациентов с ожирением и предиабетом (табл. 8) [42][ 43].

**Table:** Таблица 8. Семаглутид/Тирзепатид: влияние на риск развития сахарного диабета 2 типа у больных с ожирением и предиабетом Цель терапии: нормализация уровня гликемии, снижение массы тела

Параметр	Семаглутид 2,4 мг (STEP-10)	Тирзепатид 15 мг (SURMOUNT-1)
Характеристика пациентов	207 участников, возраст 53±11 лет, ИМТ 40,1±6,9 кг/м², HbA1c 5,9%±0,3	1032 участников, возраст 48,2±11,8, ИМТ 38,8±7,10 кг/м², НbA1c 5,8 ± 0,34 %
Изменение массы тела от исх.	-14%	-23%
Нормализация гликемии, % пациентов	81%	95%

## САХАРНЫЙ ДИАБЕТ 2 ТИПА У ПАЦИЕНТОВ С ОЖИРЕНИЕМ

Оба препарата эффективны для контроля гликемии и снижения веса у пациентов с СД2 и сопутствующим ожирением (табл. 9). Тирзепатид 15 мг показал более выраженное снижение гликированного гемоглобина (HbA1c) (–2,08% vs –1,6%) и большую долю пациентов, достигших целевых значений. Однако следует отметить, что увеличение дозы семаглутида с 1,0 мг до 2,4 мг также способствует выраженному снижению массы тела и контролю гликемии у данной группы пациентов [44][ 45].

**Table:** Таблица 9. Семаглутид/Тирзепатид: влияние на показатели углеводного обмена у больных с сахарным диабетом 2 типа и ожирением Цель терапии: нормализация уровня гликемии, снижение массы тела

Параметр	Семаглутид (STEP 2)	Тирзепатид (SURMOUNT-2)
Снижение HbA1c	–1,6%	–2,08%
Доля пациентов с HbA1c <7%	78,5%	84%
Доля с нормогликемией (HbA1c <6,5%)	67,5%	79%
Доля с нормогликемией (HbA1c <5,7%)	Не сообщается	49%
Гипогликемия	5,7% пациентов	5% пациентов
Тяжелая гипогликемия	1 случай	0 случаев

В арсенале российских врачей появились 2 препарата, которые обеспечивают выраженное снижение массы тела и контроль коморбидных состояний. В связи с этим возникает вопрос персонификации их выбора для лечения пациентов с ожирением и избыточной массой тела, оценки эффективности проводимых мероприятий. Решение этих непростых задач и легло в основу научной дискуссии экспертов, включая:

1) определение групп пациентов с ожирением для персонифицированного назначения тирзепатида /семаглутида 2,4 мг;

2) выбор терапии тирзепатидом /семаглутидом 2,4 мг с учетом профилактики сердечно-сосудистых осложнений;

3) алгоритм перевода с семаглутида на тирзепатид.

## ВОПРОС 1: ОПРЕДЕЛЕНИЕ ГРУПП ПАЦИЕНТОВ С ОЖИРЕНИЕМ ДЛЯ ПЕРСОНИФИЦИРОВАННОГО НАЗНАЧЕНИЯ ТИРЗЕПАТИДА / СЕМАГЛУТИДА 2,4 МГ

Выбор семаглутида или тирзепатида должен основываться на оценке следующих ключевых параметров (рис. 1, 2):

- исходных антропометрических показателей,

- целей по снижению массы тела,

- предыдущего опыта терапии,

- структуры коморбидности.

**Figure:**
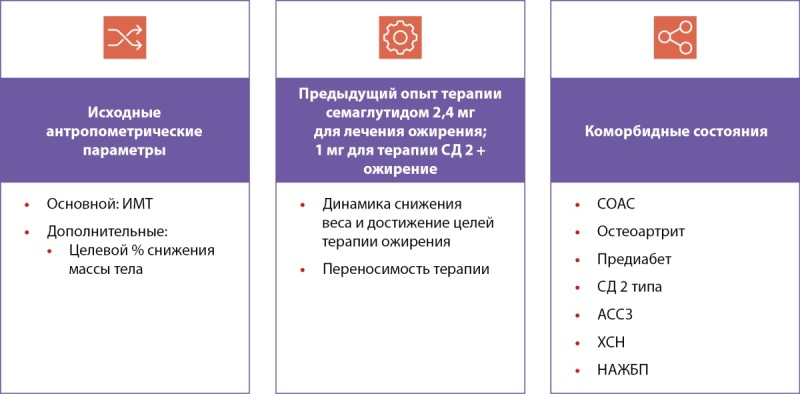
Рисунок 1. Параметры, влияющие на выбор стартового препарата.

**Figure:**
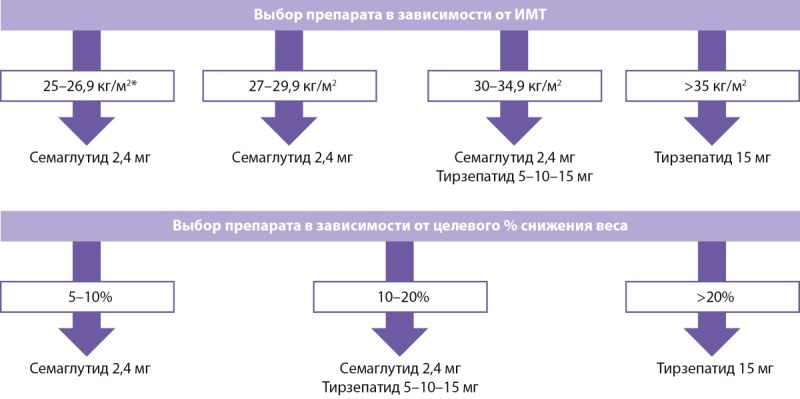
Рисунок 2. Исходные антропометрические параметры Примечание: *ИМТ 25–27 кг/м² при наличии осложнений, таких как АГ, дислипидемия, НАЖБП, СОАС, предиабет и СД2 — согласно клиническим рекомендациям ожирение 1 стадия.

ИМТ 27–29,9 кг/м² (при наличии сопутствующих заболеваний: АГ, дислипидемия, НАЖБП, СОАС, предиабет, СД2): стартовая терапия — семаглутид 2,4 мг.

Цели по снижению массы тела:

Предыдущий опыт терапии и переносимость семаглутида (рис. 3):

**Figure:**
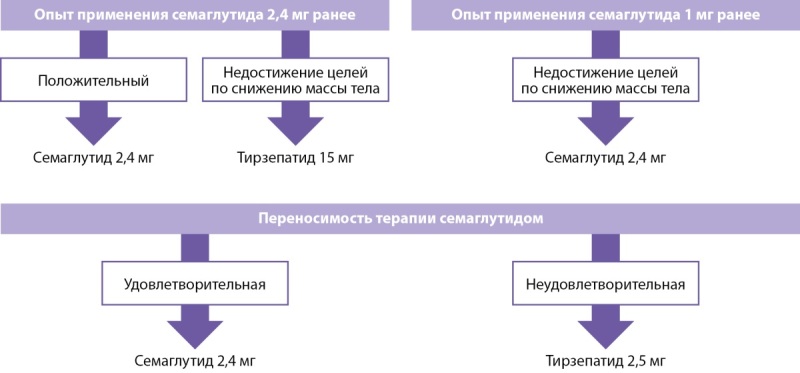
Рисунок 3. Выбор препарата для лечения ожирения в зависимости от предыдущего опыта применения или переносимости семаглутида.

Оценка эффективности терапии рекомендуется через 3 месяца после достижения максимальной терапевтической дозы (2,4 мг для семаглутида, 15 мг для тирзепатида). Минимальная рекомендуемая длительность терапии при хорошей переносимости составляет не менее 2 лет.

Выбор терапии с учетом коморбидных состояний

Наличие специфических коморбидных состояний определяет предпочтение в выборе препарата с наибольшей доказательной базой для конкретной патологии (рис. 4):

**Figure:**
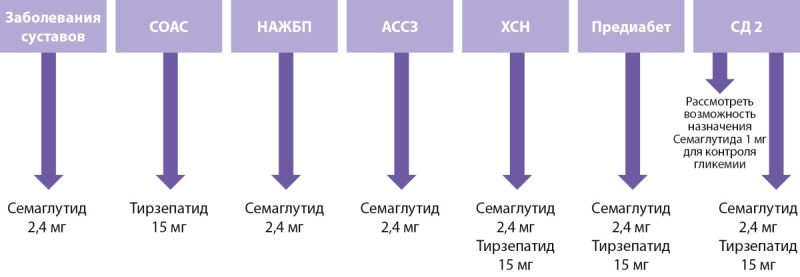
Рисунок 4. Выбор препарата для лечения ожирения в зависимости от коморбидных состояний.

Следует отметить, что несмотря на доказанные преимущества тирзепатида у пациентов с СД2, увеличение дозы семаглутида с 1,0 мг до 2,4 мг может являться приоритетной рекомендацией, учитываю его статус списке ЖНВЛП и доступность.

## ВОПРОС 2: ВЫБОР ТЕРАПИИ ТИРЗЕПАТИДОМ/СЕМАГЛУТИДОМ С УЧЕТОМ ПРОФИЛАКТИКИ СЕРДЕЧНО-СОСУДИСТЫХ ОСЛОЖНЕНИЙ

Назначение семаглутида 2,4 мг является приоритетной рекомендацией у пациентов с ожирением и сердечно-сосудистыми заболеваниями (ССЗ), поскольку он является единственным препаратом для лечения ожирения, обладающим официально зарегистрированным показанием для снижения риска основных неблагоприятных сердечно-сосудистых событий (MACE), что доказано результатами масштабного исследования SELECT: на фоне терапии было зафиксировано достоверное снижение риска MACE на 20%, сердечно-сосудистой смертности на 15% и смерти от всех причин на 19%.

## ВОПРОС 3: АЛГОРИТМ ПЕРЕВОДА С СЕМАГЛУТИДА НА ТИРЗЕПАТИД

Перевод пациента с одного препарата на другой должен осуществляться в рамках подхода «шаг за шагом» (рис. 5).

**Figure:**
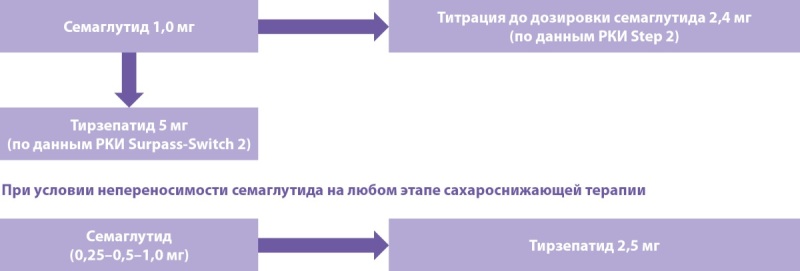
Рисунок 5. Алгоритм перехода с семаглутида 2,4 мг на тирзепатид у пациентов с ожирением + СД2.

## МОНИТОРИНГ ЭФФЕКТИВНОСТИ И БЕЗОПАСНОСТИ

Оценка эффективности терапии должна быть комплексной и включать:

## ЗАКЛЮЧЕНИЕ

## ДОПОЛНИТЕЛЬНАЯ ИНФОРМАЦИЯ

Источники финансирования. Работа выполнена по инициативе авторов без привлечения финансирования.

Конфликт интересов. Авторы декларируют отсутствие явных и потенциальных конфликтов интересов, связанных с содержанием настоящей статьи.

Участие авторов. Все авторы одобрили финальную версию статьи перед публикацией, выразили согласие нести ответственность за все аспекты работы, подразумевающую надлежащее изучение и решение вопросов, связанных с точностью или добросовестностью любой части работы.
